# Transoral settotomy for Zenker diverticulum: long-term outcomes in elderly patients

**DOI:** 10.1186/1471-2318-11-S1-A54

**Published:** 2011-08-24

**Authors:** M Rottoli, S Siboni, D Bona, I Russo, L Bonavina

**Affiliations:** 1 Department of General Surgery, University of Milan, Policlinico San Donato IRCCS, San Donato Milanese, Italy

## Background

Zenker diverticulum is often observed in elderly patients complaining of dysphagia and regurgitation. Endoscopic transoral stapling was introduced in the 90’s as an alternative to standard open diverticulectomy and cricopharyngeal myotomy for the treatment of Zenker diverticulum. Aim of this study was to evaluate the results of this procedure in patients over 75 years of age.

## Material and methods

Between 2002-2010, 73 patients (45 males, median age 74 years) underwent transoral stapling. The operation was performed under general anesthesia using a Weerda diverticuloscope and one or more cartridges of linear endostapler. Since 2008, a retention suture (Endostitch^®^) was routinely used to improve traction of the septum inside the stapler jaws. Patients OLDER and YOUNGER than 75 years were compared for perioperative variables and long-term outcomes using Chi-squared test, Student’s *t* test and Kaplan Meier analysis with log-rank test. Recurrence was defined as a relapse of the symptoms requiring reoperation.

## Results

Results are shown in Table 1. Median age of the two groups was respectively 80 (range 75-88) and 66.5 years (range 45-74). The groups were similar regarding gender, BMI, operative technique, operative time and postoperative complication rate. Only one (3%) postoperative complication occurred in the OLDER group (periesophageal abscess), and was treated conservatively. After a similar follow-up time (45 vs 48.5 months), the two groups had a comparable rate of patients complaining of regurgitation (6.1% vs 7.5%, p=0.8) and dysphagia (6% vs 10%, p=0.5). Satisfaction rate was 81.8% and 80%, respectively (p=0.8). Five year recurrence rate was 11.6% in OLDER and 10.2% in YOUNGER patients (p=0.8). The median time of recurrence was significantly longer in OLDER patients (21 vs 9.5 months, p=0.04).

**Table 1:** Comparison of short and long-term outcomes in the two groups.

Variable	OLDER (33)	YOUNGER (40)	P value
	N (%) or median (range)		

Median age (years)	80 (75-88)	66.5 (45-74)	<0.001
Male gender	17 (51.5%)	28 (70%)	0.1
Median BMI (Kg/m^2^)	23.5 (16-30)	25 (20-29)	0.9
Median diverticulum size (cm)	3 (1-6)	2.5 (1-6)	0.2
Diverticulum size < 3 cm	8 (24.2%)	21 (52.5%)	0.01
Median operative time (min)	30 (15-90)	20 (15-60)	0.2
Use of Endostitch	13 (32.5%)	14 (42.4%)	0.4
Postoperative complication	1 (3%)	0	0.3
Median follow-up time (months)	45 (5-108)	48.5 (5-104)	0.8
Long-term regurgitation	2 (6.1%)	3 (7.5%)	0.8
Long-term dysphagia	2 (6%)	4 (10%)	0.5
Satisfaction rate	27 (81.8%)	32 (80%)	0.8
5 year recurrence rate	11.6%	10.2%	0.8
Median time to recurrence (months)	21 (18-36)	9.5 (8-12)	0.04

## Conclusions

Transoral settotomy is safe and effective even in elderly patients, with short and long-term outcomes similar to younger patients. Because of the low incidence of postoperative complications and the higher rate of long-term satisfaction, the technique should be proposed as the first approach for the treatment of Zenker diverticulum in elderly patients.

